# TET2 Function in Hematopoietic Malignancies, Immune Regulation, and DNA Repair

**DOI:** 10.3389/fonc.2019.00210

**Published:** 2019-04-02

**Authors:** Yimei Feng, Xiaoping Li, Kaniel Cassady, Zhongmin Zou, Xi Zhang

**Affiliations:** ^1^Department of Hematology, Xinqiao Hospital, Army Medical University, Chongqing, China; ^2^State Key Laboratory of Trauma, Burns and Combined Injury, Army Medical University, Chongqing, China; ^3^Irell and Manella Graduate School of Biological Sciences of City of Hope, Duarte, CA, United States; ^4^Henry E. Riggs School of Applied Life Sciences, Keck Graduate Institute, Claremont, CA, United States; ^5^Department of Chemical Defense, School of Preventive Medicine, Army Medical University, Chongqing, China

**Keywords:** TET2, SIRT1, DNA demethylation, DNA repair, DNA stability

## Abstract

Over the last decade, investigation of *Ten-Eleven Translocation 2* (*TET2*) gene function and *TET2* mutation have become of increasing interest in the field of hematology. This heightened interest was sparked by the seminal discoveries that (1) *TET2* mutation is associated with development of hematological malignancies and that (2) the TET family of proteins is critical in promoting DNA demethylation and immune homeostasis. Since then, additional studies have begun to unravel the question “Does TET2 have additional biological functions in the regulation of hematopoiesis?” Here, we present a mini-review focused on the current understanding of TET2 in hematopoiesis, hematological malignancies, and immune regulation. Importantly, we highlight the critical function that TET2 facilitates in maintaining the stability of the genome. Based on our review of the literature, we provide a new hypothesis that loss of TET2 may lead to dysregulation of the DNA repair response, augment genome instability, and subsequently sensitize myeloid leukemia cells to PARP inhibitor treatment.

## Introduction

In 2009, expression of the *Ten-Eleven Translocation-2* (*TET2*) gene and its variants was demonstrated in myeloid malignancies (1). Followup studies demonstrating that proteins of the TET family play a key role in DNA hydroxymethylation further fueled interest in investigation of TET2 function in both basic and clinical research (1, 2). Here we present a review on the current understanding of TET2 in hematopoiesis and hematopoietic malignancies, as well as discuss the disputed clinical prognosis of *TET2* mutation. Moreover, we highlight the role of TET2 in DNA damage and repair, and provide evidence to support the hypothesis that TET2 interacts with other DNA repair-related proteins to maintain genome stability. We further propose that loss of TET2 may sensitize myeloid leukemia cells to DNA repair stress, such as is induced by treatment with PARP inhibitors.

## TET2 and its Enzymatic Activity

*TET2* gene, a member of the TET family of enzymes, is located on chromosome 4q24, and its protein product TET2 modulates DNA hydroxymethylation by converting 5-methylcytosine (5 mC) to 5-hydroxymethylcytosine (5 hmC) to promote DNA demethylation (3). TET enzymes can modify 5 mC through oxidation, a phenomenon which revealed an alternative pathway for DNA demethylation mechanisms. It is noted that *TET2* mutations are consistently associated with a decrease in 5hmC, which has been suggested as a potential diagnostic and prognostic biomarker for hematopoietic malignancies, especially myeloid malignancies (4).

**Figure 1:**
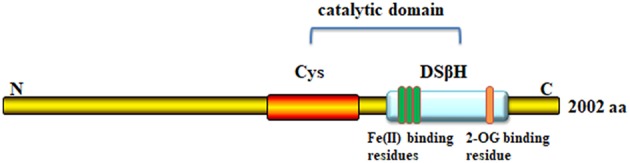
Domain structure of TET2 protein. The C-terminal core catalytic domain consists of a cysteine (Cys)-rich domain, and double-stranded β-helix fold domain (DSβH domain), each of which contains three Fe-binding domains and one site for 2-oxoglutarate binding.

The TET2 functional domain is at the C-terminus, consisting of a cysteine (Cys)-rich domain and a double-stranded β-helix fold (DSβH) domain (Figure 1). The DSβH domain contains the important metal-binding residues for Fe (II)/α-KG (also known as 2-oxoglutarate). TET dioxygenases require oxygen, α-KG and Fe (II) for their activity (5). TET uses molecular oxygen as a substrate to catalyze the oxidative decarboxylation of α-KG, which generates enzyme-bound Fe (IV)-oxo, and subsequently converts 5 mC to 5 hmC (6, 7).

## TET2 and Its Partners

**Figure 2:**
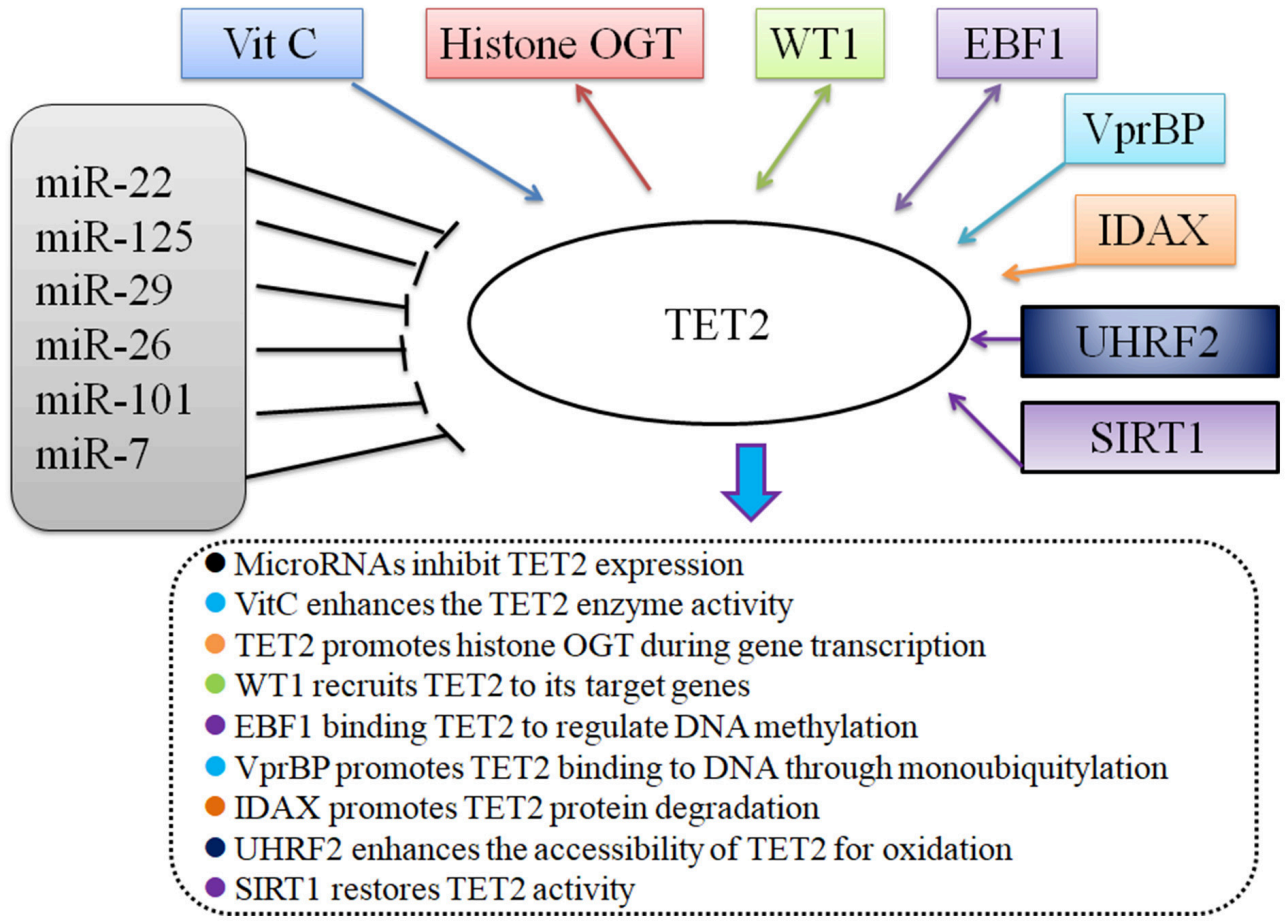
TET2 and its partners.

Since the discovery of TET2, numerous studies have focused attention on the enzymatic effects of the TET2 protein, presumably through its function in DNA demethylation. TET2-interacting proteins have been identified and analyzed by expressing tagged-TET2, followed by affinity purification coupled with mass spectrometry. Known TET2 binding partners include small molecules, micro-RNAs, and some transcription factors (8). TET2 can recruit O-linked β-D-N-acetylglucosamine (O-GlcNAc) transferase (OGT) to chromatin, an effect that is independent of its enzymatic activity. TET2 and OGT colocalize on chromatin, increase H3K4me3 level, and regulate gene transcription (9, 10). Additionally, multiple studies have confirmed that microRNAs (miR-7, miR-22, miR-26, miR-29, miR-101, miR-125) (11) negatively regulate TET2 gene expression at the pre-mRNA level (8, 12). Vitamin C can restore and enhance TET2 enzymatic activity to suppress leukemia (13, 14). Furthermore, several proteins, such as WT1 (15, 16), VprBP (17), EBF1 (18), IDAX (19), and UHRF2 (20), are reported to exert their biological functions in combination with TET2 (5). Sun et al. recently reported sirtuin 1 (SIRT1) deacetylates TET2 in myelodysplastic syndrome stem and progenitor cells; moreover, SIRT1-deficient MDS HSPCs (CD34^+^ cells) exhibit enhanced cell growth and self-renewal (21). In summary, TET2 expression is tightly regulated at the pre-translational level and the TET2 protein is believed to exert its function via TET2-containing protein complexes (Figure 2). However, additional TET2 partners remain to be identified.

## Roles of TET2 in Hematopoiesis and Hematopoietic Malignancies

TET2 plays important roles in hematopoiesis, including promoting the self-renewal of stem cells and lineage commitment and terminal differentiation of monocytes (5). TET2 is highly expressed in hematopoietic stem/progenitor cells, and the deletion of *TET2* in primary bone marrow cells leads to the increase of the percentage of immature c-Kit^+^Lin^−^ cells, suggesting that loss of TET2 may affect stem/progenitor cell differentiation (4, 22). *TET2* deletion in CD34^+^CD38^+^ cells can promote monocyte expansion, indicating a regulatory role of TET2 in lineage commitment (23, 24).

**Figure 3:**
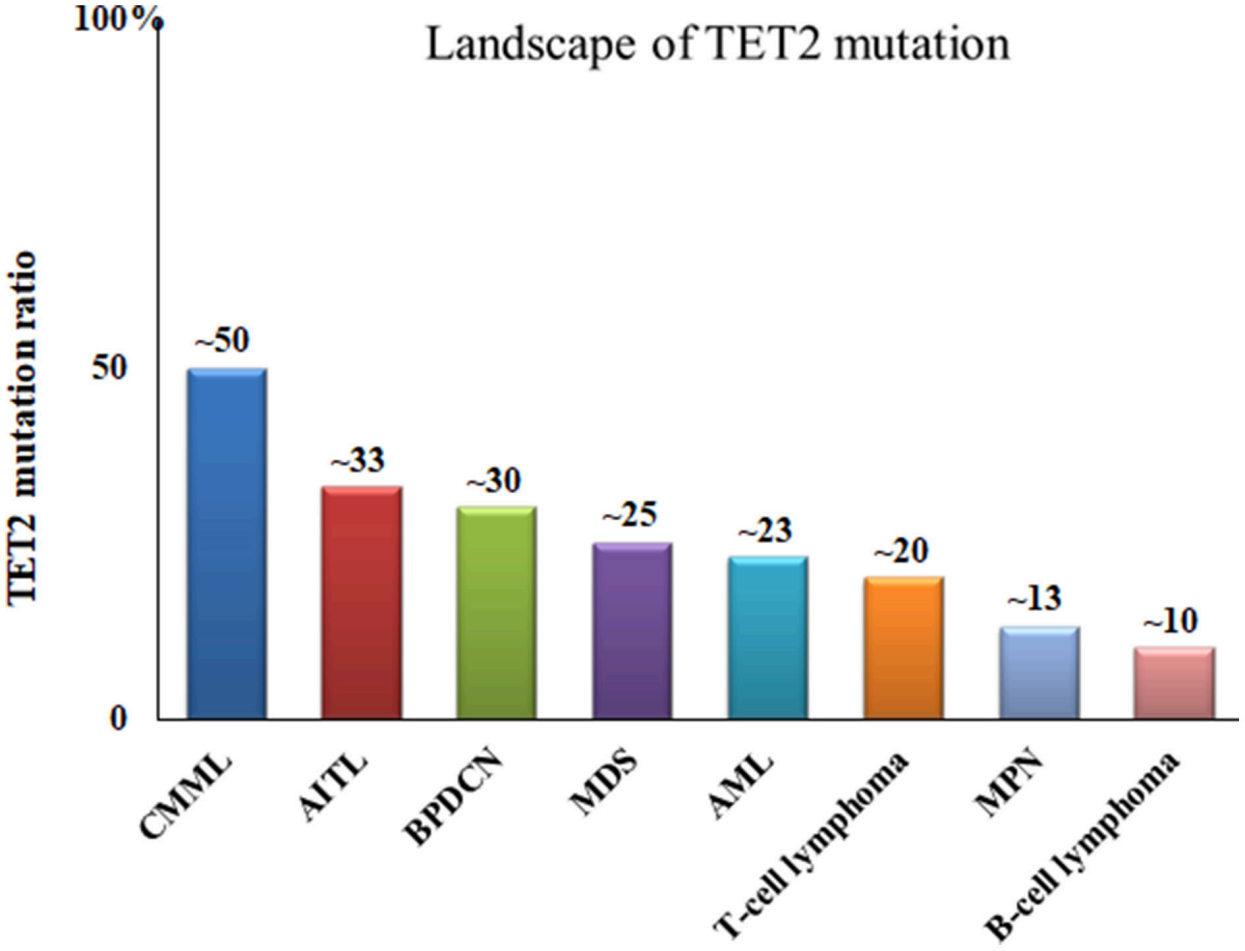
Frequencies of *TET2* gene mutations in malignant blood disease. CMML, chronic myelomonocytic leukemia; AITL, angioimmunoblastic T-cell lymphoma; BPDCN, blastic plasmacytoid dendritic cell neoplasm; MDS, myelodysplastic syndromes; AML, acute myelogenous leukemia; MPN, myeloproliferative neoplasms.

*TET2* has been widely recognized as a tumor-suppressor gene. *TET2* deletion is sufficient to cause myeloid and lymphoid malignancies in mice (25). Homozygous and heterozygous mutations in the *TET2* gene are recurrent events in human hematopoietic malignancies. In a range of myeloid and lymphoid neoplasms, the frequencies of *TET2* mutations are 20–35% in myelodysplastic syndrome (MDS) (26), 30–60% in chronic myelomonocytic leukemia (CMML) (27), 12–34% in acute myeloid leukemia (AML) (28) and 2–33% in lymphoid malignancies (5) (Figure 3).

*TET2* gene mutations include frame shift, generated stop codons, in-frame deletion, and amino acid substitutions of highly conserved residues. As for the incidence of homozygous or heterozygous mutations in given disease patterns, such as AML, CMML and MDS, there is no specific genotypic pattern. Viguie et al. (29) analyzed six patients with deletions or mutations of *TET2* in myeloid cancers, and reported that five patients had a heterozygous loss, and one had a deletion of both *TET2* copies (1). Solary et al. (5) reported the highest rate of *TET2* genetic alterations was observed in CMML in which a heterozygous or homozygous mutation was identified in 50–60% of this overlapping MDS/MPN; however prognosis associated with prevalence of heterozygous vs. homozygous mutation was not reported.

Genomic sequencing of all coding *TET2* exons revealed those homozygous mutations tend to be associated with uniparental disomy 4q, and the heterozygous genotype is associated with lack of chromosomal lesions (30). Heterozygous *TET2* knockout in mice, which led to ~50% loss of *TET2* gene expression, resulted in significant but slower and less frequent malignant transformation than double-allele knockout (25). The 5-hydroxymethylcytosine level in DNA was reduced dramatically in homozygous *TET2*-mutant mice compared to heterozygous *TET2*-mutant mice. Approximately 33% of homozygous *TET2*-mutant and 8% of heterozygous *TET2*-mutant mice developed lethal myeloid malignancies in the first year of life (25). However, heterozygous and homozygous mutations of *TET2* were found in patients with similar clinical manifestations and disease phenotype, and Kaplan-Meier curves suggested there was also no difference on Overall Survival (OS) between the homo/hemizygous and heterozygous cases (30). On the contrary, patients with homozygous *TET2* mutation showed significantly inferior Event-Free Survival (EFS) and a higher relapse rate compared with those with heterozygous *TET2* mutation (31). Thus, while homozygous *TET2* deletion in murine models conveys increased susceptibility to malignancies, *TET2* genotype relative to patient outcome in human malignancies is less clear (see below).

## Prognosis of *TET2* Mutations in Myeloid Malignancies

When it comes to prognosis of myeloid malignancies with *TET2* mutations, the effect of *TET2* mutations remains controversial (Table 1) (39). Those mutations include missense, non-sense, and frame shift mutations spanning the entire *TET2* coding sequences (28). Large cohort studies showed that *TET2* mutations did not impact the overall survivals in AML (32, 33) and myeloproliferative neoplasma (MPN) patients (34). On the other hand, some reports found that *TET2* mutant-CMML (35) and -AML (36, 37, 40) patients had poorer outcomes compared with patients without *TET2* mutations. In contrast, *TET2* mutations have been shown to confer superior survival in MDS (26, 38). Furthermore, *TET2* mutations may predict a more favorable response to hypomethylating agents (HMAs) in high-risk patients (41–43). Two meta-analyses (44, 45) suggest that *TET2* mutations have no prognosis impact on OS of patients with MDS. However, low expression of *TET2* is clearly associated with an unfavorable prognosis in MDS patients. Because these reports are clinical retrospective studies lacking the support of basic, preclinical scientific research, the possible underlying mechanisms that account for the prognosis of *TET2* mutations in different malignancies are unknown. Whether *TET2* mutations dysregulate pathways already known to contribute to hematopoietic transformation, or represent a novel pathway to transformation, remains to be elucidated (36).

**Table 1:** Prognosis of TET2 mutations in myeloid malignancies.

**Prognostic relevance**	**Patients studied**	**Diseases**	**References**
Not impact on overall survival	111 patients with *de novo* AML	AML	(32)
	247 patients with AML derived from MDS or therapy related AML	Secondary AML	(33)
	239 BCR-ABL-negative MPN patients	MPN	(34)
Shorter disease-free and overall survival	88 patients with CMML	CMML	(35)
	119 AML patients including *de novo* AML, therapy related AML, and AML with an antecedent hematologic disorder	AML	(36)
	427 patients with normal karyotype AML	AML	(37)
Improved survival and a lower risk of transformation to AML	89 MDS patients and 7 with MDS transformed to AML	MDS	(26)
	153 Chinese patients with MDS	MDS	(38)

**Figure 4:**
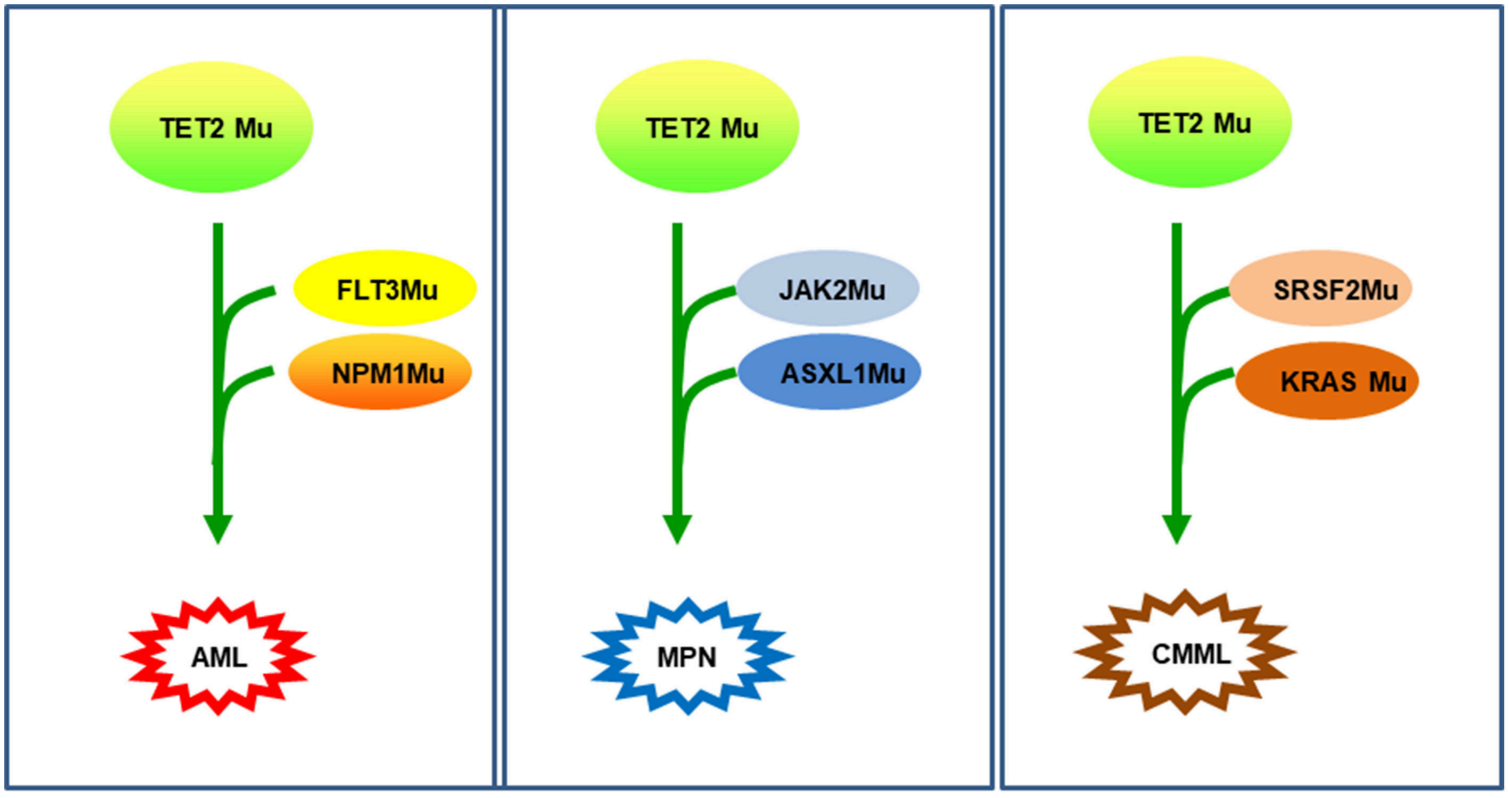
*TET2* mutations as background mutations. Thanks to *TET2* mutations as background mutations, the disease will occur and develop by the accumulation of additional mutations. In the first example (42), TET2-D1384V in HSCs, then in *NPM1* and *FLT3* mutation, induced an AML. In the second example (31, 44), *TET2* mutation with JAK2V617F-positive generated thrombocythemia, which can transform into secondary myelofibrosis due to an additional *ASXL1* mutation. In the third example (23), mutations in *SRSF2*, then in *KRAS* mutation developed a chronic myelomonocytic leukemia (CMML).

*TET2* mutations, as an early event in pathogenesis, may cooperate with other gene mutations, deemed background mutations, to promote various hematological malignancies. For example, *TET2* mutations, when harboring *FLT3-ITD* mutation, induced AML (46); *TET2* mutations, if combined with *JAK2* and *ASXL1* mutation, generated MPN, such as polycythemia vera (PV) and secondary myelofibrosis (MF) (47–50); finally, *TET2* mutations, together with mutations in *SRSF2* and *KRAS*, were associated with CMML (23) (Figure 4). It is obvious that different combinations of *TET2* mutations along with other gene mutations will inevitably predict different prognoses. With the use of new drugs, such as HMAs, the clinical prognosis of *TET2* mutation may also be improved.

## Roles of TET2 in Immune Regulation

**Figure 5:**
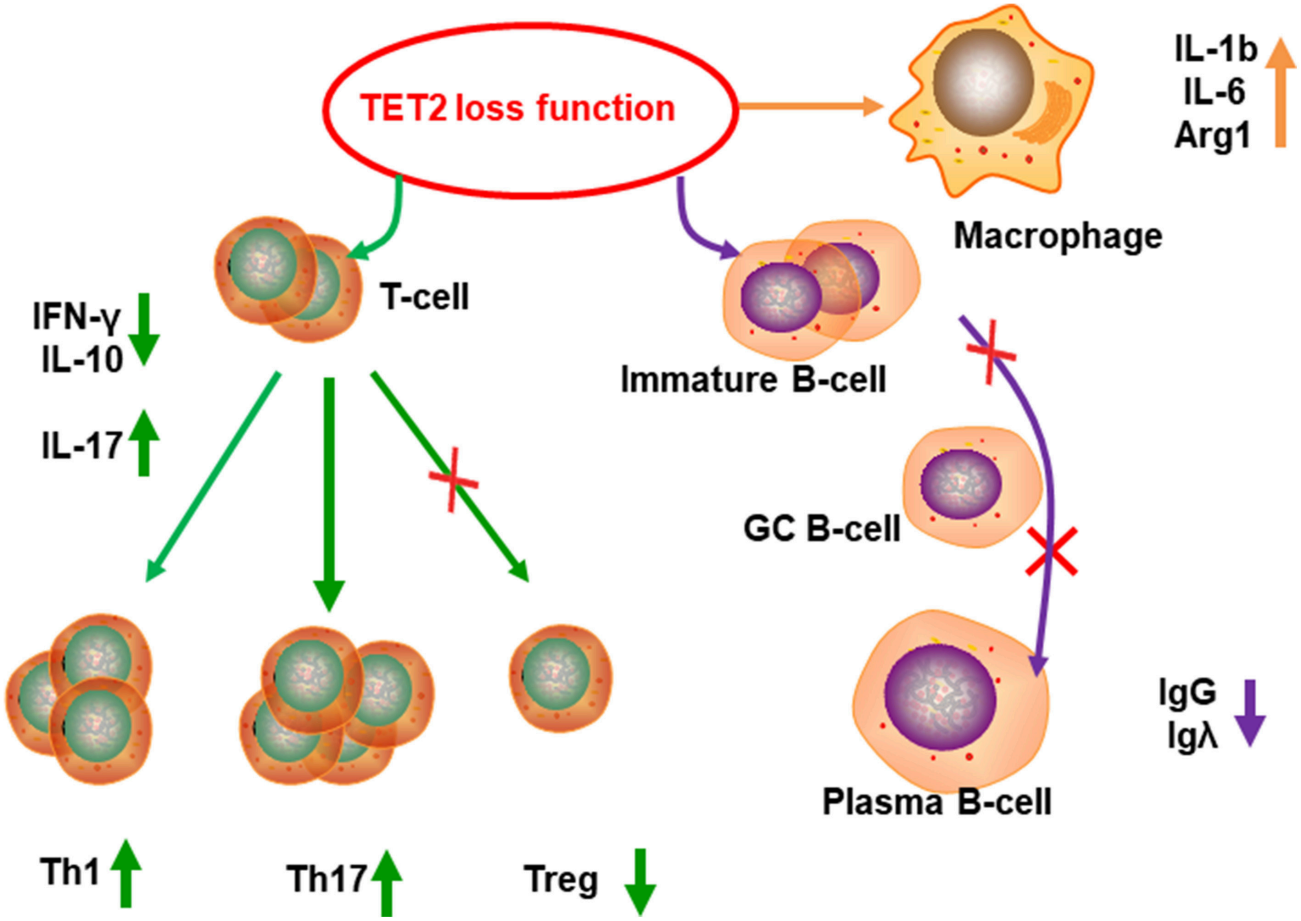
TET2 functions in immune regulation. TET2 can regulate the T cell differentiation. Because of TET2 loss of function, population of Treg cells decreased, Th1 and Th17 subsets increased, cytokines secretion changed, such as increased IL-17, and reduction of IL-10 and IFN-γ. TET2 deficiency causes germinal center hyperplasia, impairs plasma cell differentiation, restrains IgG and Igλexpression, and promotes B-cell lymphomagenesis. TET2 can inhibit inflammation in MΦs, TET2^−/−^ MΦs produce more inflammatory factor, such as IL-1b, IL-6, and arginase1.

Recently, it was reported that TET2 also participated in regulation of the immune system (51). TET2 is highly expressed in each T helper cell (Th cell) subset compared with the other TET family genes. TET2^−/−^ T cells showed a marked reduction in IFN-γ and IL-10 production at both the mRNA and protein level, which was associated with reduced p300 recruitment, suggesting TET2 may regulates Th1 and Th17 cell differentiation (52). In a murine model of multiple sclerosis, (autoimmune encephalomyelitis-EAE) model, upregulation of TET2 inhibited naive CD4^+^ T cell proliferation and differentiation into Th1 and Th17 cells and was largely protective against development of EAE (53). In another study, it was shown that TET2 mutation, cooperating closely with RhoA, leads to abnormal CD4^+^ T cell proliferation and disruption of T cell homeostasis (54). TET2 also can mediate *Foxp3* demethylation to drive T regulatory cell (Treg) differentiation and maintain immune homeostasis (55). TET2 also plays a role in the regulation of the innate immune system and was found to be highly expressed during murine macrophage (MΦ) differentiation. TET2 loss increased IL-1b, IL-6, and Arginase1 mRNA expression, indicating that TET2 can restrain inflammation mediated by murine MΦs (56) (Figure 5). *TET2/TET3* conditional knockout at early stages of B-cell development largely prevents lineage-specific programmed demethylation events, thus causing defects in B-cell differentiation and function (57). *TET2* deficiency results in germinal center hyperplasia, impairs plasma cell differentiation, and promotes B-cell lymphomagenesis (58). Interestingly, introduction of anti-CD19 chimeric antigen receptors (CARs) occasionally disrupts the *TET2* base sequence (59). Consequently, *TET2*-disrupted CAR T cells were shown to display a central memory phenotype and induced long-term leukemia remission (59). *TET2*-deficient macrophages altered the tumor microenvironment to reduce tumor burden during melanoma progression (60). In a separate study, TET2-deficient CD8^+^ tumor infiltrating lymphocytes (TILs) also displayed increased anti-tumor efficiency in a mouse model of melanoma (61). In summary, TET2 plays a critical role in maintaining and/or establishing immune tolerance. Thus, manipulation of TET2 function may be possible for the promotion of tolerance (such as during autoimmunity) or in the promotion of antitumor immunity.

## Roles of TETs in DNA Damage and Repair

**Figure 6:**
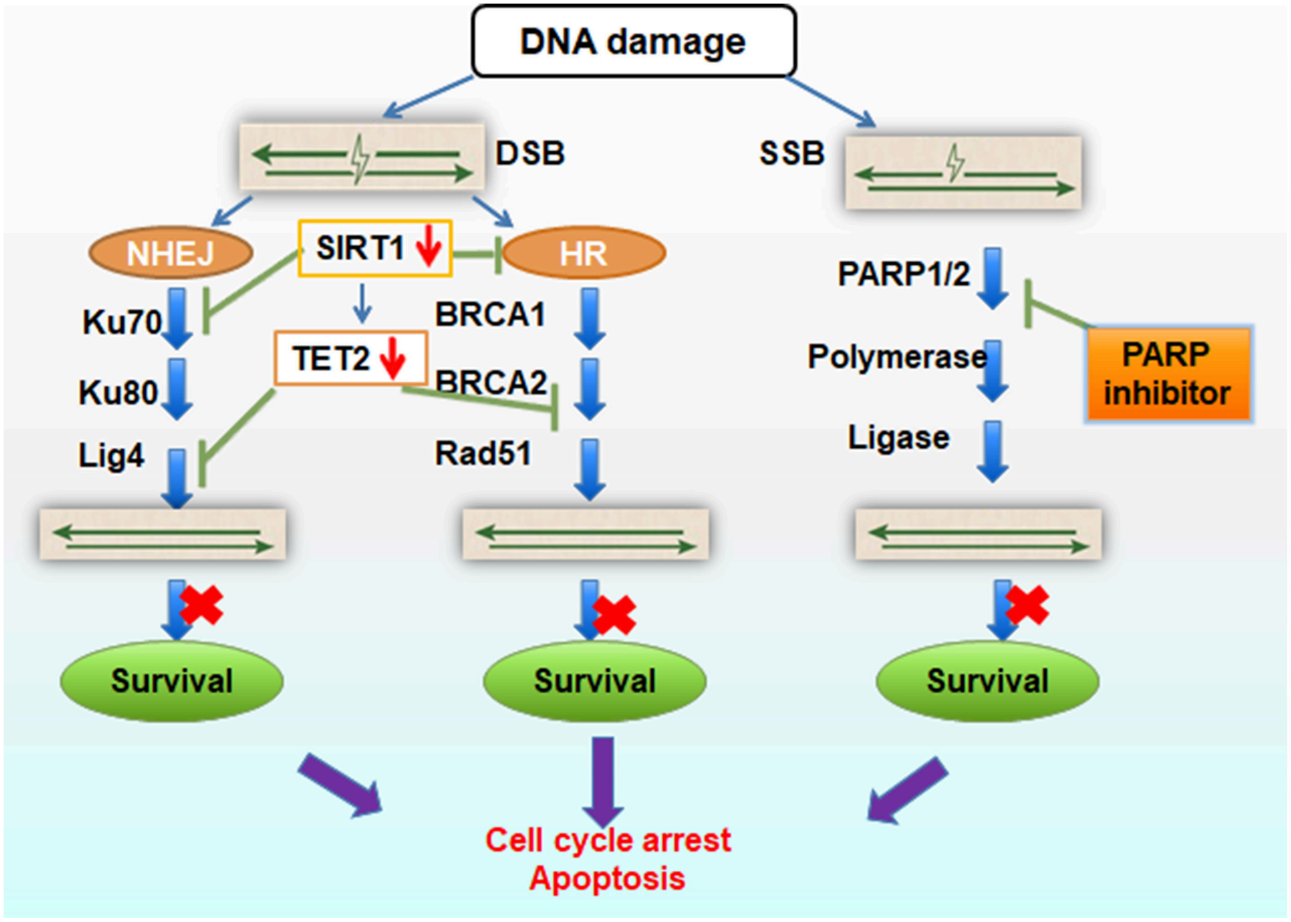
Schematic diagram of TET2 function in DNA repair. TET2 knock-down results in lower expression of Lig4 and BRCA2 expression, and SIRT1 deficiency results in Ku70 and TET2 inactivation, blocking the NHEJ and HR repair. If combined with inhibition of PARP inhibitor in SSB repair, the whole DNA repair pathways are harmed, cell survival gets impeded, and cell apoptosis is elevated.

DNA strand damage is primarily sub-divided into double-strand breaks (DSB) and single strand breaks (SSB). DSBs are the most hazardous of all types of DNA damage; when unrepaired, DSBs can be lethal and trigger cellular apoptosis. There are two major pathways for DSB repair, non-homologous end joining (NHEJ) and homologous recombination (HR). Ku70, Ku80, and Lig4 are involved in NHEJ, while BRCA1/BRCA2, RAD51 are involved in the HR pathway. PARP1/PARP2 participates in SSB repair, and PARP (poly(ADP-ribose) polymerase) inhibition causes PARP-1 to be trapped onto DNA repair intermediates, especially during base excision repair of SSB (Figure 6).

In fact, the Tet family (Tet1/2/3) is reported to be involved in the DNA damage and repair response pathways. TET1 deficient cells harbored significantly more DNA strand breaks even in the absence of exogenous DNA damaging agents (62). TET1 may be involved in protecting cells against more severe injuries, such as DNA strand breaks. TET1 regulates several important DNA repair genes via modulating H4K16ac at the promoter regions of DNA repair genes including RAD50, BRCA1, RAD51, and TP53BP1 (63). Loss of TET1 leads to DNA instability and resulted in the development of myeloid malignancy in mouse models (64, 65). Additionally, Tet3-mediated conversion of 5 mC to 5 hmC promotes ATR-dependent DNA damage response and regulates DNA repair (66). Although the biochemical properties of TET2 have been extensively studied, the effect of TET2 loss-of-function in DNA damage repair is less understood. Recently, Kafer et al. reported that 5 hmC is actively enriched at endogenous DNA damage sites in cancer cell lines, precisely where TET2 creates damage-associated 5 hmC foci (67). Deficiency of TET2 elicits chromosome segregation defects in response to DNA replication stress. It has also been reported that *TET2* knockout can decrease BRCA2 mRNA expression (67). As previously mentioned, BRCA1/BRCA2 proteins are important in maintaining genomic stability, especially in HR during the DNA damage response. It is reported that wild-type TET2 induction resulted in an increased level of 5-hmC and a cell cycle defect in S phase associated with higher level of phosphorylated P53, a process which is tightly controlled to avoid genetic and chromosomal instabilities (68). Taken together, it is reasonable to suppose that TET2 can maintain genomic stability via promotion of the DNA damage repair.

## Hypothesis and Supportive Evidence

Based on our review of the literature, we hypothesize that loss of TET2 might sensitize myeloid leukemia cells to poly (ADP-ribose) polymerase inhibitors (PARPis). PARPis have become a mainstay for treatment of certain malignancies, especially breast and ovarian cancers (69), because they can selectively target tumor cells with BRCA1/2 mutation or HR deficiency (70). In CML (71), fanconi anemia (FA) (72) and some therapy-related AML samples (73, 74), BRCA1 or/and BRCA2 mutations have been found and suggested to be pathogenic genotypes, which may serve as models for PARPis application to leukemia therapy. Moreover, several clinical studies have reported that PARPis are effective in leukemia treatment (75, 76), but the detailed mechanism underlying their effectiveness needs to be fully explored. As mentioned earlier, TET2 deletion also leads to downregulation of BRCA2 gene expression—a phenomenon which directly supports the underlying principle of our hypothesis. It is important to highlight that reduction of BRCA function as a result of TET2 deletion may not be equivalent to loss-of-function resulting from BRCA mutation. However, it is reported that BRCA reduction can also sensitize tumor cells to PARPis. For example, miR-182-mediated downregulation of BRCA1 impacts DNA repair and sensitivity to PARPis (77). MiR-9 mediates the downregulation of BRCA1, impedes DNA damage repair in ovarian cancer and improves chemotherapeutic efficacy by increasing the sensitivity of cancer cells to DNA damage (78). PI3K inhibition impairs BRCA1/2 expression and sensitizes BRCA-proficient triple-negative breast cancer to PARP inhibition (79). Additionally, BRCA-deficient tumors are hypersensitive to DNA damaging chemotherapeutic agents such as cisplatin, mitomycin C, etc. (80, 81). Accordingly, loss of TET2 results in BRCAs reduction, impairs homologous recombination in DNA repair, and, if combined with DNA damaging chemotherapeutic agents, might sensitize tumor cells to PARPis.

Secondly, TET2 plays a particular role in the NHEJ pathway of DNA repair. Wang et al. (15) over-expressed TET2 in HL60 cells, and subsequently found that expression of Lig4 mRNA increased 4-fold. Because Lig4 is critical for DNA repair through NHEJ pathway (82), loss of TET2 might decrease the Lig4 recruitment, result in DNA repair failure, and cell death. In our own studies, we found that SIRT1 can bind to TET2 and restore its activity (21). Overexpressing SIRT is reported to activate TET2 function, which suggests that SIRT1 may provide a novel therapeutic target in MDS (83, 84). These published data suggest that SIRT1 might interact with TET2 to promote DNA stability. It is reported that active SIRT1 promotes genomic stability and that SIRT1 specifically activates Rad51-independent HR (85). On the other hand, SIRT1 promotes DNA repair activity in NHEJ by the deacetylation of Ku70 (86). Thus, SIRT1 insufficiency could feasibly restrain function of TET2, and disable the DNA DSB repair. BRCAs, Lig4 and SIRT1, as partner proteins of TET2, are closely related to NHEJ and HR pathway of DNA repair.

Taken together, we propose that loss of TET2 can inhibit its partner proteins function (such as BRCAs, Lig4, SIRT1) in DSB DNA repair. If combined with PARPis to block the SSB repair pathway, cells are prone to cell cycle arrest and apoptosis (Figure 6). The experiments required to test our hypothesis is relatively straight-forward. For example, our hypothesis can be tested by treating TET2KO cells with PARPis, such as AZD2281 (Olaparib) and MK4827 (Niraparib), two selective inhibitors of PARP1/2, and analyzing cellular function/viability through cytotoxicity assays and apoptosis detection. On the other hand, rescue experiments, knocking in and overexpressing TET2, can further test whether TET2 overexpression provides protection from PARPis. Finally TET2^−/−^ AML mice model and/or clinical patients‘ samples with TET2 mutation also can be treated with PARPis to observe the treatment's effect and further explore the mechanism of TET2 in regulating genomic stability.

## Conclusions and Future Direction

TET2 plays important roles in epigenetic regulation, stem cell differentiation, and development of hematopoietic malignancies. Loss of TET2 function leads to DNA hypermethylation and subsequent dysregulated gene expression in hematopoietic stem cells, and has been considered as an initial step of myeloid malignant transformation including MDS and AML. While enzymatic activity of TET2 is well studied, little is known about other biological functions of TET2, for example, as a scaffold protein to recruit partners in DNA damage repair. Furthermore, the prognosis of TET2 mutation in hematologic malignancies has been controversial and the detailed mechanism of TET2 in promotion of malignancy needs to be further explored. In general, malignancies expressing the TET2 mutation are sensitive to treatment with HMAs. However, as some malignancies are resistant to HMAs, investigating additional drugs for the treatment of patients with TET2 mutation expressing malignancies is of dire importance. Due to the role of TET2 in promoting DNA stability, PARPis represent a class of drugs that offer potential effectiveness in treating HMA-resistant myeloid neoplasms. Additional studies are required to shed light on the potential therapeutic role of disrupting TET2 pathway to augment genome instability for the treatment of cancer.

## Author Contributions

XL, KC, XZ, ZZ, and YF: conceptualization; XZ and YF: funding acquisition; XL, XZ, ZZ, and YF: methodology; YF: wrote original draft and revised the manuscript; YF, KC, ZZ, and XZ: writing-review and editing.

### Conflict of Interest Statement

The authors declare that the research was conducted in the absence of any commercial or financial relationships that could be construed as a potential conflict of interest.

## Abbreviations

TET2: Ten-Eleven Translocation 2
HMAs: hypomethylating agents
SIRT1: sirtuin 1
MDS: myelodysplastic syndrome
OS: overall survivals
PARP: poly (ADP-ribose) polymerase
BRCA: breast cancer susceptibility (BRCA) genes
HR: homologous recombination
NHEJ: non-homologous end-joining.
